# Factors affecting COVID-19 vaccine uptake in populations with higher education: insights from a cross-sectional study among university students in Malawi

**DOI:** 10.1186/s12879-024-09534-3

**Published:** 2024-08-21

**Authors:** Qebo Kornelio Madhlopa, Matthews Mtumbuka, Joel Kumwenda, Thomas Arron Illingworth, Marie-Claire Van Hout, Joseph Mfutso-Bengo, Chomora Mikeka, Isaac Thom Shawa

**Affiliations:** 1 Kamuzu University of Health Sciences, P/Bag 360, Chichiri Blantyre 3, Blantyre, Malawi; 2 Faculty of Science, University of Malawi Chancellor College, P.O. Box 280, Zomba, Malawi; 3 UbuntuNet Alliance, Onions Office Complex, Off Mzimba Street, P.O. Box 2550, Lilongwe, Malawi; 4 Research, Innovation and Impact, South East Technological University, Waterford, Cork Road Campus, X91 K0EK Ireland; 5 School of Human Science, University of Derby, Kedleston Road, Derby, DE22 1GB UK

**Keywords:** COVID-19, Vaccine uptake, Vaccine hesitancy, Vaccine resistance, Malawi, University students, Health messaging, Misinformation, Disinformation

## Abstract

**Background:**

The Coronavirus disease-2019 (COVID-19) vaccines were rolled out in many countries; however, sub-optimal COVID-19 vaccine uptake remains a major public health concern globally. This study aimed at assessing the factors that affected the uptake, hesitancy, and resistance of the COVID-19 vaccine among university undergraduate students in Malawi, a least developed country in Africa.

**Methods:**

A descriptive cross-sectional study design was conducted using an online semi-structured questionnaire. A total of 343 University undergraduate students in Blantyre participated in this study after obtaining ethical clearance. Data was exported from Survey Monkey to Microsoft Excel version-21 for cleaning and was analysed using SPSS version-29. Descriptive statistics, including percentages, were performed to define the sample characteristics. Pearson Chi-square and Fisher’s exact test were performed to identify significant relationships between vaccine uptake and demographics. A 95% confidence interval was set, and a p-value of < 0.05 was considered statistically significant.

**Results:**

Of the 343 participants, 43% were vaccinated. Among the vaccinated, the majority (47.3%, *n* = 69/146) received Johnson & Johnson vaccine followed by AstraZeneca (46.6%, *n* = 68/146). The commonly reported reason for vaccine acceptance was ‘to protect me against getting COVID-19’ (49%); whereas vaccine hesitancy was attributed to ‘lack of knowledge (34%), and concerns about vaccine safety (25%).

**Conclusions:**

This study found that adequate knowledge about benefits and safety of COVID-19 vaccine could potentially increase uptake. Lack of credible information or misinformation contributed to vaccine hesitancy. The findings provide insights for design of strategies to increase future vaccine uptake and reduce determinants of vaccine hesitancy. To reduce vaccination hesitancy in any population with or without higher education, we recommend that institutions entrusted with vaccine management must optimise health messaging, and reduce mis-information and dis-information.

## Introduction

In late 2019, a novel coronavirus (CoV) epidemic was reported in Wuhan city, Hubei Province, China following suspected cases who presented with various degrees of pneumonia-like symptoms of unknown aetiology. The majority of cases reported a history of exposure to the Huanan seafood market (popularly known as ‘wet market’), that prompted the Wuhan local health authority to close the market down, apply disinfection measures, and institute a vigorous case finding and identification [1–4]. The virus that caused the outbreak was called the 2019 novel coronavirus (2019-nCoV) by the World Health Organization (WHO) [5, 6] on 11th February 2020. The 2019 novel coronavirus was designated a *severe acute respiratory syndrome coronavirus 2* (SARS-CoV-2) by the International Committee on Taxonomy of Viruses following identification [3, 7, 8]; and subsequently became referred to as the coronavirus disease 2019 (COVID-19) [9, 10]. On 9th January 2020 a full genetic sequence of the new virus was made available by a team of Chinese investigators [3, 11], it shared 79.5% of the genetic sequence of the SARS-CoV that caused the 2002–2003 pandemic [12].

In terms of the existing evidence base on SARS-CoV, between 2002 and 2003, SARS-COV first emerged in Guangdong, China [13, 14], the virus rapidly spread across 29 countries, infecting more than 8000 people that resulted in 10% mortality rate [15]. In contrast, in 2012, Middle East respiratory syndrome coronavirus (MERS-CoV) appeared in Saudi Arabia [16] where infected people presented similar symptoms to SARS-CoV; but MERS-CoV had a much higher mortality rate of 34% [17]. On 30th January 2020, the WHO declared the COVID-19 a public health emergency of international concern (PHEIC). Previously, the WHO declared six other PHEICs namely: SARS-CoV (2003 in China), H1N1 (2009), Polio (2014), Ebola (2014 in West Africa), Zika (2016 in Brazil) [18, 19], and Ebola (2019 in the Democratic Republic of Congo) [20, 21]. Until early December of 2019, only six different CoVs (SARS-CoV) [22], MERS-CoV [23], HCoV-NL63, HCoV-229E, HCoV-OC43 and HKU1) were known to infect humans presenting different clinical features than range from mild common cold-like symptoms to severe respiratory illness [7]. The HCoV-229E and HCoV-OC43 were isolated in the 60s [24–26] whereas the HCoV-NL63 and HCoV-HKU1 strains were identified in the 2000s following the SARS-CoV outbreak [27, 28].

In terms of transmission and spread of disease, the SARS-CoV-2 is zoonotic but human-to-human transmission through contact with infected respiratory droplets is one the highest risk factors. The COVID-19 general incubation period takes 14 days even longer [29]. First studies in Wuhan estimated an average incubation time of 5.2 days [5], 3.0 days [29], and 6.4 days [30]. The CoVs cause multiple systemic infections mainly respiratory distress similar to SARS and Middle East respiratory syndrome (MERS) infections [22, 23, 31]. The SARS-CoV-2 rapidly spread from China to the rest of the world causing great panic and threat to humanity. Following on from the initial reported cases, reports of cases began to be reported around the world. National governments announced public health emergencies and instigated various public health campaigns and disease mitigation initiatives to reduce the spread of infections. This included lockdowns, wearing face masks, social/physical distancing, handwashing with sanitizers, and travel restrictions [32].

As of the end of 2022, 50 different COVID-19 vaccines were rolled out globally. Despite this, vaccine uptake was affected by multi-faceted factors such as misinformation, myths, perception of acceptance, knowledge of the disease and its outcomes [33–35], attitudes and beliefs towards vaccination, perceived risks and severity to infection, vaccine characteristics, advice and information from healthcare officials and relatives, general health related behaviors, and vaccine accessibility and affordability of vaccines [36].

In Malawi, the first case was identified on 2nd April 2020 occurring mainly among travelers, and those who had contact with travelers from the hotspot regions including China. Following first case identification, increased rates of new infections were subsequently reported across the country mainly among travelers from hotspot regions including China, secondary transmission, tertiary and quaternary transmissions were observed despite strict preventive and control measures. Noting this, the Malawi government embarked on national vaccination campaigns to reduce disease severity and curb the outbreak. However, vaccine hesitancy coupled with a short expiration date of the donated vaccines led to large quantities of COVID-19 vaccines to be discarded in an incinerator on May 19, 2021 [37]. Very little is documented about the Malawi COVID-19 vaccination experience. Our study responds to this gap, and was conducted to assess key determinants of COVID-19 vaccine uptake, hesitancy or resistance among tertiary education students in Malawi.

## Materials and methods

A cross-sectional web-based survey was conducted in 2022 among Kamuzu University of Health Sciences (KUHeS) students in Malawi utilizing a self-administered electronic questionnaire designed and hosted on survey monkey software in English language. KUHeS is a public institution of higher learning formed by merging College of Medicine, and Kamuzu College of Nursing (formerly under the University of Malawi). It is located in Blantyre and offers comprehensive health and allied sciences programmes.

Due to the COVID-19 restrictions across the country during the time of this study (August to November 2022), an online survey study was deemed most appropriate despite its potential to exclude some participants with no access to internet-based services. The online self-administered questionnaire was developed by the research team and partially adapted from the Healthwatch may 2021 Template survey questions for COVID-19 [38]. Further considerations were applied following guidance from a previous study by Geldsetzer et al. [39].

Of the 1800 enrolled students a sample size of 327 was calculated using the Slovin’s formula under a margin of error of 0.05%; with a 95% confidence interval, a standard normal variate (z score) of 1.96, and an estimated proportion of 0.5 (or percentage of the population) [40]. A convenience sampling approach was applied where undergraduate students from KUHeS were invited to participate in the study following a written consent. Study participants were asked to complete the online questionnaire utilizing platforms such as WhatsApp® and E-mail. Posters containing the participant information sheet with a QR code link to the survey were posted around the university campuses notice boards to inform and recruit participants. Students were encouraged to pass on the questionnaire to their colleagues. Automated notifications were sent to the students every two weeks in order to remind them of their participation in the survey. Students who experienced challenges with unavailability of internet-connected devices or intermittent connectivity, were provided with the researchers’ devices to support the completion of the survey. The opening page of the questionnaire contained the study information, aim and objectives, and a participant’s consent section.

All study participants acknowledged being 18 years or older; and provided a written consent for inclusion before their participation in the study. Information such as gender, religion, vaccination history, willingness to be vaccinated was collected in order to assess factors that contribute to vaccine hesitancy, uptake or resistance. Ethical permission was obtained from College of Medicine Research Ethical Committee (Reference number: U.11/21/3510).

The data was cleaned and coded using Microsoft excel; and analyzed using SPSS version 29 (SPSS@IBM). Microsoft Excel was used to code all the themes into numerical variables that were exported to SPSS for statistical analysis. Descriptive statistics, including percentages, were performed to define the sample characteristics. In addition, Pearson Chi-square and Fisher’s exact test were performed to identify significant relationships between vaccine uptake and demographics. A 95% confidence internal was set, and a p-value of < 0.05 was considered statistically significant.

## Results

343 undergraduate students participated in the study. The majority (87.2%, *n* = 299/343) were aged 18–24 years old, the rest aged above 25. Of the total study participants, 90.4% (*n* = 310/343) belonged to Christianity, 7.0% (*n* = 24/343) Islamic, 0.9% (*n* = 3/343) Hinduism religions, and 1.7% (*n* = 6/343) were non-religious (Table 1). The study respondents were studying Medicine and Surgery (MBBS), Medical Laboratory Sciences (MLS), Pharmacy, Basic Medical Sciences (BMS), Physiotherapy, Dentistry, Nursing, and Dietetics and Human Nutrition (Table 1).

Overall, 42.6% (*n* = 146/343), of the participants had received at least one dose of the COVID-19 vaccine, whereas 57.4% (*n* = 197/343) did not. Among the vaccinated, the majority (47.3%, *n* = 69/146) had received Johnson & Johnson vaccine, 46.6% (*n* = 68/146) received AstraZeneca, whereas Pfizer, Sinopharm BIBP, Sinovac vaccines were given to 2.7% (*n* = 4/146), 1.4% (*n* = 2/146), and 0.7% (*n* = 1/146) of the vaccinated participants respectively. The other 1.4% (*n* = 2/146) of the vaccinated participants did not provide details of the type of COVID-19 vaccine they had received (Table 1). Sinopharm BIBP, and Sinovac vaccines were not available in Malawi at the time of the study, but the respondents were international students who had received them in their respective countries.

**Table 1:** Demographic details and vaccination status of the study participants

	Number of Participants (*n* = 343)	Percentage
**Gender**		
Male	199	58.0%
Female	144	42.0%
**Age (yrs)**		
18–24	299	87.2%
25–30	37	10.8%
31–34	3	0.9%
35–40	4	1.2%
**Religion**		
Christianity	310	90.4%
Islam	24	7.0%
Hinduism	3	0.9%
Non-religious	6	1.7%
**Vaccination status**		
Vaccinated	146	42.6%
Not vaccinated	197	57.4%
**Type of vaccine received**		
AstraZeneca	68	46.58%
Johnson & Johnson	69	47.26%
Pfizer	4	2.74%
Sinopharm BIBP	2	1.37%
Sinovac	1	0.68%
Do not know	2	1.37%
**Program of study**		
Medical Laboratory Sciences	47	14%
Medicine and Surgery	165	48%
Pharmacy	38	11%
Physiotherapy	32	9%
Dentistry	15	4%
Diuretics and Human Nutrition	12	4%
Basic Medical Science	5	2%
Nursing	29	9%
**Residential status**		
On-campus	266	78%
Off-campus	77	22%
**Year of study**		
0/Premed/Foundation	82	24%
1	76	22%
2	48	14%
3	39	11%
4	70	20%

**Fig. 1:**
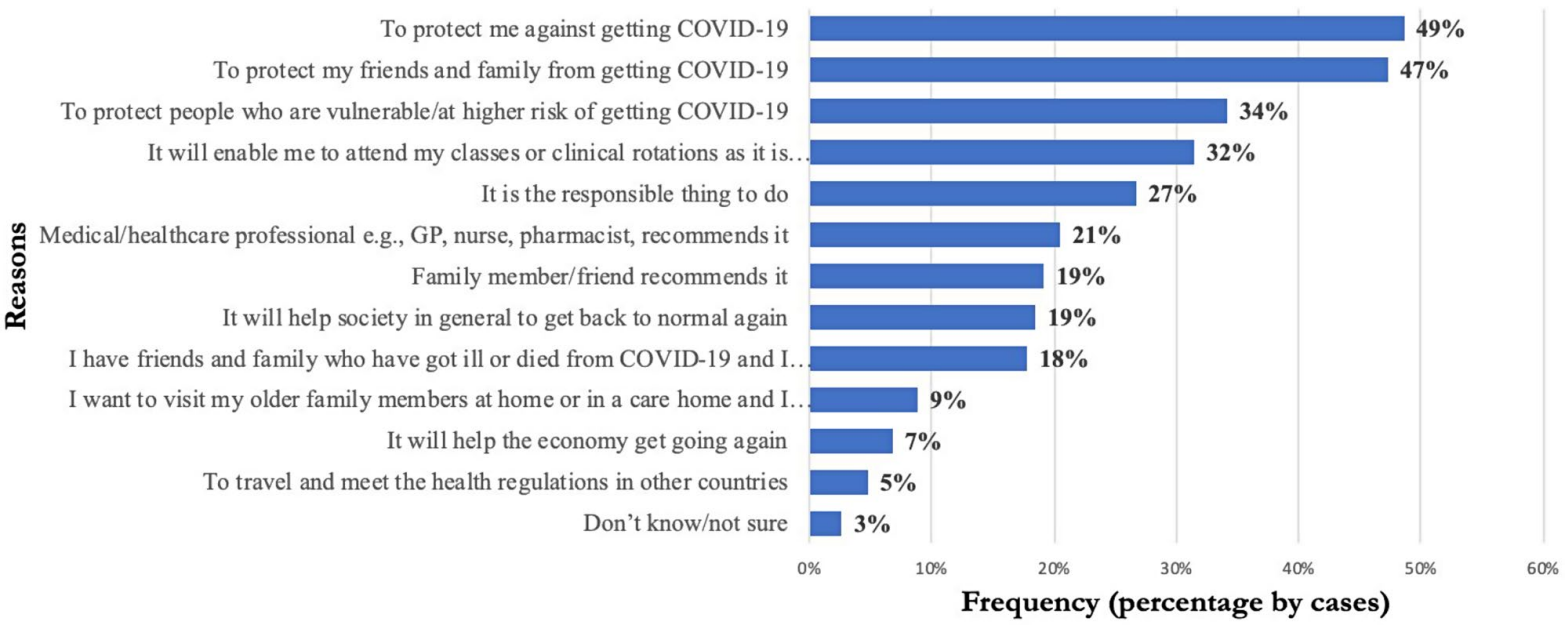
Factors influencing vaccine uptake

**Fig. 2:**
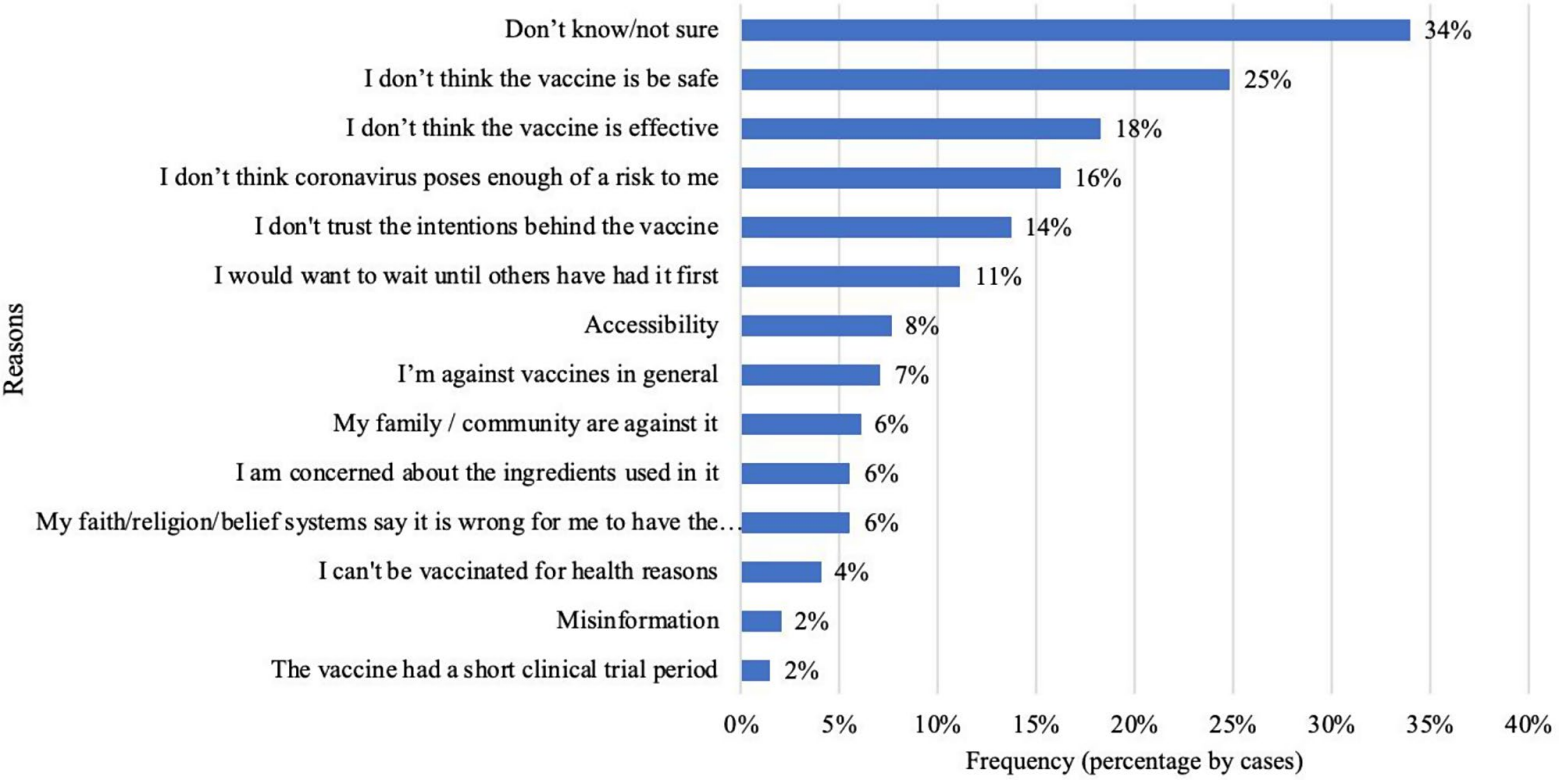
Factors influencing vaccine hesitancy

Figures 1 and 2 provide a clear summary of some of the factors that contributed to vaccine acceptance and hesitancy respectively. The commonly reported reasons for vaccine acceptance were: (1) ‘To protect me against getting COVID-19’ (49%), (2) ‘To protect my friends and family from getting COVID-19’ (47%), ‘To protect people who are vulnerable/at higher risk of getting COVID-19’ (34%), ‘It will enable me to attend my classes or clinical rotations as it is mandatory to get the vaccine’ (32%), ‘It is the responsible thing to do’ (27%), ‘Medical/healthcare professional e.g., GP, Nurse, Pharmacist recommend it’ (21%), ‘Family member/friend recommends it’ (19%), ‘It will help society in general to get back to normal again’ (19%), and ‘I have friends and family who got ill and died from COVID-19and I know how serious it is’ (18%). Only 5% gave ‘To travel and meet the health regulations in other countries’ as a reason for vaccine acceptance (Fig. 1).

The majority (34%) did not give any reason (don’t know/not sure) for vaccine hesitancy. Those that indicated a reason for vaccine hesitancy, 25% cited ‘I don’t think the vaccine is safe’ as the main reason; whereas ‘Misinformation’, and ‘The vaccine had a short clinical trial period’ were the least factors for vaccine hesitancy as cited by 2% only of the respondents (Fig. 2).

The analysis of the study participants’ demographic details that included gender, age, religion, programme of study, and their COVID-19 vaccination history were presented in a frequency table (Table 2). According to the results presented by the Chi Square test suggest an association (2 × 2) between vaccination and gender with more females (45%) reported to have received the vaccine than their male counterparts (41%) (X^2(1,343) = 0.672, *p* = 0.412). There was a significant association between vaccination and year of study with clinical students having a higher vaccination rate (74%) than pre-clinical students who had a low vaccination rate (22%) (X^2(1,343) = 90.579, p = < 0.001). The statistical analysis performed using the Fisher exact test of association suggest that there was a significant association between vaccination and age, with older age groups having a high vaccine uptake compared to younger age groups (p = < 0.001). However, there was no significant association between vaccination and religion (*p* = 0.246) (Table 2).

**Table 2:** Participants’ demographics and vaccine uptake associations

Demographics	Vaccinated participants (*n* = 146)	Unvaccinated participants (*n* = 197)	Total (Rows)	Significance of association
**Gender**				
Female	45% (65)	55% (79)	100% (144)	*P* = 0.412
Male	41% (81)	59% (118)	100% (199)	
**Age**				
35–40	75% (3)	25% (1)	100% (4)	*P* = < 0.001
31–34	0% (0)	100% (3)	100% (3)	
25–30	68% (25)	32% (12)	100% (37)	
18–24	39% (188)	61% (181)	100% (299)	
**Religion**				
Religious	43% (145)	57% (192)	100% (337)	*P* = 0.246
Non-religious	17% (1)	83% (5)	100% (6)	
**Residential status**				
On-campus	47% (126)	53% (140)	100% (266)	*P* = < 0.001
Off-campus	26% (20)	74% (57)	100% (77)	
**Year of study**				
Clinical	74% (101)	26% (36)	100% (137)	*P* = < 0.001
Pre-clinical	22% (45)	78% (161)	100% (206)	

## Discussion

To the authors’ knowledge this was one of the few studies that explored the determinants of vaccine uptake, hesitancy, and resistance among tertiary education population in Malawi. Approximately 43% of the sample acknowledged to have received at least a single dose of COVID-19 vaccine; whereas 57% were not vaccinated. Willingness to be vaccinated was contributed by factors such as potential protection from COVID-19, a mandatory requirement in order to attend classes and clinical placements, a responsible thing to do, a recommendation by medical/healthcare practitioners, a recommendation by family and friends, to support the economy to get going, and to meet the travel requirements.

Religion did not play a significant role in vaccine uptake. Other studies have reported that Muslims were less likely to accept the COVID- 19 vaccine [35]. The participants aged above 25 years were more likely to accept the vaccine as opposed to those between 18 and 24 years old. This could probably be due to the risk perception of morbidity and mortality as a motivating factor for vaccine uptake. The older students were in clinical years where it was mandatory to receive the vaccine in order to be accepted into the clinical area.

Studies elsewhere on similar populations have had mixed reports of vaccine uptake among university students. Indian medical students had a COVID-19 vaccine uptake of 89%, and Maastricht University students in Netherlands reported an 80% uptake [41, 42]. Ethiopian university students have reported an uptake rate of 50.6%, and Nigerian Enugu state medical students have reported an uptake rate of 20.6% [43, 44]. There appears to be low vaccine uptake observed among African university students possibly due to misinformation or lack of knowledge. In other studies, conducted among the general population in Indonesia, United States of America, and United Kingdom, higher exposure to knowledge and affordability could potentially enhance high vaccine uptake now and in future [45–47].

As reported in other countries, among various social factors, lack of knowledge, was the highest independent determining factor for vaccine hesitancy. This study did not identify the information sources that were available to the study participants to aid their decision-making process. Other studies have reported that individuals whose source of information was the internet were more likely to refuse vaccination [48].Our findings concur with another study conducted among Russian university students which reported that to ‘protect themselves and avoid disturbances or limitations to their studies’ was a motivating factor for vaccinating [49]. Among Egyptian university students, ‘high knowledge of COVID-19 vaccine and positive beliefs about the vaccine’ were associated with COVID-19 vaccine uptake [50]. Some of the reasons for high vaccine uptake reported in other studies included: ‘perceived susceptibility to COVID-19, credible/reliable information, vaccine safety, perception of the disease being preventable by the vaccine, to boost immunity, and high knowledge [43, 51–54].

Vaccine characteristics: susceptibility to infection: and willingness to protect one self and family/friends were the main drivers of COVID-19 acceptance among Malawi university students. These factors could be potential determinants of future vaccine update, and should be considered when designing vaccine dissemination campaign messages. In this study, the participants cited ‘I don’t think the vaccine is safe’ as the main reason; whereas ‘misinformation’, and ‘the vaccine had a short clinical trial period’ were the least factors for vaccine hesitancy as cited by 2% only of the respondents.

Finally, this study observed that more students residing on campus accommodation were vaccinated compared to those residing outside university accommodation. Students in halls of residence live in communities that could have influenced their intentions to vaccinate. On the contrary, off campus residency probably hindered students to reach vaccination centers. A study in Lebanon, among university students also found residency status to be associated with vaccine hesitancy [52]. This study has observed that a larger number of clinical students were vaccinated compared to preclinical students. This could be attributed to their risk of exposure and also the vaccine being mandatory for them. This is similar to Hong Kong medical students whom clinical students were more vaccinated than pre-clinical students [53]. Even though there was no strong association, we observed that more females than males were vaccinated; more religious than non-religious were vaccinated.

There were several limitations of this study namely: the estimation of the students’ population especially in Nursing School at the time of the study, lack of sufficient questions covering social, behavioural, cultural, psychological, and emotional factors. There was a possibility of selection bias among study participants who were largely concentrated in the health and clinical sciences educational pathways. The responses represent their opinions at the time of participation in this study, and the information they had at the time.

## Conclusion

The study contributes to the small research base on COVID-19 vaccination in Malawi. Almost half of the sample were vaccinated, with vaccine hesitancy attributed to ‘lack of knowledge, and concerns about vaccine safety’. The study highlights the importance of tailored public health messages during public health emergencies. We recommend that institutions entrusted with vaccine management must optimise health messaging, and reduce mis-information and dis-information.

## Data Availability

The datasets used and/or analysed during the current study available from the corresponding author on reasonable request.

## Abbreviations

SARS-CoV-2: Severe acute respiratory syndrome coronavirus 2
2019-nCoV: novel coronavirus
CoV: Coronavirus
WHO: World Health Organization
COVID-19: Coronavirus disease 2019
MERS: Middle East respiratory syndrome
PHEIC: Public health emergency of international concern
MMR: Measles, mumps and rubella
